# Live-cell imaging of early events following pollen perception in self-incompatible *Arabidopsis thaliana*

**DOI:** 10.1093/jxb/eraa008

**Published:** 2020-01-14

**Authors:** Frédérique Rozier, Lucie Riglet, Chie Kodera, Vincent Bayle, Eléonore Durand, Jonathan Schnabel, Thierry Gaude, Isabelle Fobis-Loisy

**Affiliations:** 1 Laboratoire Reproduction et Développement des Plantes, Univ Lyon, ENS de Lyon, UCB Lyon 1, CNRS, INRAE, F-69342, Lyon, France; 2 CNRS, UMR 8198 Evo-Eco-Paleo, Université de Lille - Sciences et Technologies, Villeneuve d’Ascq, France; 5 University of Nottingham, UK

**Keywords:** Actin, Arabidopsis, compatible and incompatible pollinations, hydration threshold, mechanical pressure, pollen–stigma interaction

## Abstract

Early events occurring at the surface of the female organ are critical for plant reproduction, especially in species with a dry stigma. After landing on the stigmatic papilla cells, the pollen hydrates and germinates a tube, which penetrates the cell wall and grows towards the ovules to convey the male gametes to the embryo sac. In self-incompatible species within the Brassicaceae, these processes are blocked when the stigma encounters an incompatible pollen. Based on the generation of self-incompatible Arabidopsis lines and by setting up a live imaging system, we showed that control of pollen hydration has a central role in pollen selectivity. The faster the pollen pumps water from the papilla during an initial period of 10 min, the faster it germinates. Furthermore, we found that the self-incompatibility barriers act to block the proper hydration of incompatible pollen and, when hydration is promoted by high humidity, an additional control prevents pollen tube penetration into the stigmatic wall. In papilla cells, actin bundles focalize at the contact site with the compatible pollen but not with the incompatible pollen, raising the possibility that stigmatic cells react to the mechanical pressure applied by the invading growing tube.

## Teaser

A live imaging system coupled with self-incompatible Arabidopsis lines highlights the role of stigmatic cells in controlling pollen hydration and in reacting to pollen tube intrusion by remodeling the actin cytoskeleton.

## Introduction

Flowers of Brassicaceae species have a dry stigma, highly discriminatory with early control of pollen capture following pollination (Dickinson, 1995). The stigma consists of a dome of flask-shaped epidermal cells (papillae). Once pollen grains land on papillar cells, only those recognized as compatible are accepted, whereas undesirable pollen grains [i.e. from unrelated species or self-pollen in self-incompatible (SI) species] are rejected (reviewed in Doucet *et al.*, 2016). Pollen grains need to hydrate on the stigma so as to permit the emergence of a pollen tube that penetrates the papilla cell wall and then grows within the pistil tissues (reviewed in Chapman and Goring, 2010). *In vitro* assays showed that growing through the stigma is required for pollen tubes to efficiently target the ovules (Palanivelu and Preuss, 2006). Likewise, genetic ablation of the stigmatic papillae prevents normal pollen hydration or germination (Tung *et al.*, 2005). Early events of the pollen–stigma interaction are thus crucial steps for successful fertilization.

Most of the Brassicaceae species have a self-incompatibility system, which allows the stigma to reject self-pollen grains and hence to prevent self-fertilization (de Nettancourt, 2001). This self/non-self recognition mechanism, genetically controlled by a single multiallelic locus, the *S*-locus, depends on a receptor–ligand interaction (Ivanov *et al.*, 2010). The ligand, the *S*-locus cysteine-rich protein (SCR/SP11), is present in the material contained in the cavities of the pollen cell wall (Schopfer *et al.*, 1999; Takayama *et al.*, 2000), flows out onto the papilla surface, and interacts with the *S*-locus receptor kinase (SRK) (Stein *et al.*, 1991; Iwano *et al.*, 2003), localized to the plasma membrane of stigmatic papillae. This interaction induces SRK phosphorylation and activation of the signaling cascade, leading to self-pollen rejection (Cabrillac *et al.*, 2001). Incompatible pollen grains fail to hydrate properly and do not germinate or only germinate a short pollen tube whose growth is arrested on papilla cells (Dickinson, 1995; Hiroi *et al.*, 2013). Although *Arabidopsis thaliana* is self-fertile, transgenic SI *A. thaliana* lines were generated by reintroducing SCR/SP11 and SRK gene pairs from its close SI relative *Arabidopsis lyrata* (Nasrallah and Nasrallah, 2002; Nasrallah *et al.*, 2004). SI Arabidopsis lines have opened the way for thorough analysis of pollen–stigma recognition mechanisms through the use of the genetic tools and mutant collections available for this model plant species.

Although they have been studied for many years, the early steps leading to the pollen acceptance or rejection decision are still not clearly characterized. In this work, based on the generation of new SI Arabidopsis lines, we revisited the cellular aspects of pollen–stigma interaction by setting up a live imaging system to monitor pollen hydration and germination following compatible as well as incompatible pollinations. We quantified the degree of pollen hydration required for pollen activation and highlighted a late self-incompatibility control restricting penetration of the stigmatic cell wall by the pollen tube. Furthermore, we monitored the dynamics of the actin network during compatible and incompatible pollination and found that remodeling of actin cables is triggered in stigmatic cells only when the pollen–stigma interaction is engaged in compatibility.

## Materials and methods

### Plant growth conditions

*Arabidopsis thaliana* Col-0 and C24, *A. lyrata* ssp. *petraea* haplotype *S14* originating from the Czech Republic (seeds kindly provided by Dr Pierre Saumitou-Laprade, Université de Lille, F59655 Villeneuve d’Ascq cedex, France), and all transgenic plants were grown in growth chambers under a long-day cycle of 16 h light/8 h dark at 21 °C/19 °C with a relative humidity of ~60%. The *A. lyrata* plants were propagated by cuttings every 3 months.

### *A. lyrata SCR14* and *SRK14* gene cloning

The *AlSRK14* genomic sequence (3620 kb) was amplified with specific AttB-containing primers (Supplementary Table S1 at *JXB* online) and subsequently inserted by BP recombination into a pDon207 plasmid. A fragment of 4081 kb containing the *AlSCR14* gene was amplified using the AttB-containing primer (Supplementary Table S1). This fragment was subsequently inserted by BP recombination into a pDONR-Zeo plasmid.

Sequences of *AlSRK14* and *AlSCR14* transgenes introduced in *A. thaliana* have been submitted to GenBank (*pSLR1-gAlSRK14*, accession number MH680585; *pgAlSCR14*, accession number MH680584).

### Plasmid construction and plant transformation

We used Gateway^®^ vectors (Life Technologies, USA; http://www.thermofisher.com/, Karimi *et al.*, 2002) for expression of transgenes in *A. thaliana.* The DNA fragment containing the *Brassica oleracea SLR1* stigma-specific promoter (1.5 kb upstream of the *SLR1* start codon, Hackett *et al.*, 1996; Fobis-Loisy *et al.*, 2007) was inserted into the pDONP4-P1R plasmid (Durand *et al.*, 2014). Two pollen-specific promoters were used, either *pLAT52* from *Solanum lycopersicum* (Twell *et al.*, 1989; Rotman *et al.*, 2003) or *pACT11* from *A. thaliana* (Huang *et al.*, 1997; Rotman *et al.*, 2005), which were inserted by recombination into the plasmid pDONP4-P1R. The coding sequences (CDS) of TURQUOISE (TURQ), green fluorescent protein (GFP), or red fluorescent protein (RFP) were introduced into pDONP2R-P3 (+Stop). The sequence encoding *LTI6b* (Cutler *et al.*, 2000) was amplified and inserted in the pDon207 plasmid. The entry clone containing the LifeAct peptide fused to VENUS was provided by Takashi Ueda (Era *et al.*, 2009).

Expression vectors were produced by three fragment LR recombination. The *SLR1* promoter, the genomic sequence of *AlSRK14*, and a 3' mock sequence were inserted in the pK7m34GW destination vectors. The genomic sequence of *AlSCR14* [including the promoter and 3' untranslated region (UTR)], a 5' mock sequence, and a 3' mock sequence were inserted in plasmid pB7m34GW. The *SLR1* promoter, the *LifeActin:Venus*, and a 3' mock sequence were inserted in pB7m34GW (Act:Venus). The *LAT52* promoter, the TURQ CDS (+stop), and a 3' mock sequence were inserted in pK7m34GW. The *ACT11* promoter, the *RFP* CDS (+stop), and a 3' mock sequence were inserted in the plasmid pK7m34GW. The *SLR1* promoter, the *LTI6b* sequence, and the *GFP* CDS (+stop) were inserted in the plasmid pB7m34GW.

Arabidopsis transgenic plants were generated using *Agrobacterium tumefaciens*-mediated transformation according to Logemann *et al.* (2006). The *AlSRK14* construct was introduced into Col-0 and C24. *AlSCR14* and *LAT52-*TURQ constructs were introduced into C24. The Act:Venus construct was introduced into the selected *AlSRK14* line #10. The *pACT11-RFP* construct was introduced in the selected *AlSCR14* line #4. *LTI6b:GFP* was introduced into Col-0. For all transformations, unique insertion lines homozygous for the transgene were selected by segregation on antibiotic-containing medium

### Reverse transcription–quantitative real-time PCR

Thirty stigmas from stage 13 to early 14 (14E) *A. thaliana* flower buds or 20 stigmas from *A. lyrata* buds just before anthesis were dissected and total RNA was extracted with the Arcturus^®^ PicoPure^®^ RNA isolation kit (Life Technologies). A 265 ng aliquot of total RNA was reverse transcribed with random hexanucleotides and RevertAid (200 U µl^–1^; Thermo Scientific), and subjected to quantitative real-time PCR with *SRK14*-specific primers and primers within the *ACTIN8* gene used as an internal control. Because the *AlSRK14* expression level was compared between *A. lyrata* and *A. thaliana*, we designed primers that amplify a region of the *ACTIN8* cDNA conserved between the two species (Supplementary Table S1). Amplification of SRK14 was performed with two primers located within the first exon (Supplementary Table S1). Absence of contaminating genomic DNA was controlled with primers located within a non-coding region close to the *ACTIN8* gene (Supplementary Table S1). Quantitative analysis of real-time PCR results was performed using the 2^–ΔΔCt^ method (Schmittgen and Livak, 2008) and normalized to the *ACTIN8* reference.

### Pollination assays

Flower buds at the end of developmental stage 12 (Smyth *et al.*, 1990) were emasculated and 18 h later stigmas that had reached developmental stage 13 or 14E were manually pollinated with mature pollen. Six hours after pollination, stigmas were fixed in 10% acetic acid, 50% ethanol (v/v) , and stained with aniline blue (Kho and Baër, 1968) for pollen tube counting. In a second series of pollination assays, stage 12 flower buds were emasculated and, 42 h (stage 15) or 66 h (stage 16) later, stigmas were pollinated with fresh mature pollen for 6 h followed by fixation and aniline blue staining. In a third experiment, stigmas were pollinated at stage 13–14E and collected after 6, 24, 48, or 72 h followed by fixation and aniline blue staining. Pollination assays were carried out on at least three stigmas and repeated on at least two different dates. Pollination was considered as incompatible when fewer than five pollen tubes were counted in the stigma (Kitashiba *et al.*, 2011).

### Dual pollination: semi-*in vivo* assay

Stage 12 flower bud was emasculated, and 18 h later the (stage 13–14E) pistil was cut transversally in the middle of the ovary and placed vertically in a half-cut, perforated PCR tube whose base was introduced into a solid agar medium. Pollen was deposited by gently touching the stigma with a mature anther. Incompatible pollen was deposited first, followed (within <1 min) by deposition of compatible pollen, which defined the timing starting point (T0) for monitoring pollen behavior. A cover slip was delicately applied on the surface of the pollinated stigma for confocal imaging. The system was maintained throughout the experiment at 21 °C and under 45% relative humidity. To increase the relative humidity in the vicinity of the pollinated stigma, pieces of solid agar medium were added around the mounted pistil. For cell surface labeling, after 35 min in high humidity conditions, pollinated stigma was incubated in FM4-64™ dye (*N*-3-triethylammoniumpropyl-4-6-4-diethylamino phenyl hexatrienyl pyridinium dibromide, Life Technologies T3166, 8.23 μM) for 5 min and subsequently washed in half-strentgth Murashige and Skoog basal medium containing 10% (w/v) sucrose, before mounting between a slide and cover slip in this medium.

### Confocal microscopy and actin fluorescence intensity

Images were acquired with an SP8 laser-scanning confocal microscope (Leica) using a ×25 objective (numerical aperture 0.95, water immersion). VENUS and GFP were excited at 488 nm and fluorescence was detected between 510 nm and 551 nm, RFP and FM4-64 were excited at 552 nm and fluorescence detected between 598 nm and 648 nm, and TURQ was excited at 448 nm and fluorescence detected between 450 nm and 481 nm. To avoid emission spectrum overlay, image acquisition was performed with the sequential mode. Serial confocal images encompassing the entire volume of the stigma were recorded every 1 µm and every 1 min or 2 min. Images and movies were processed with ImageJ software (https://imagej.nih.gov/ij/)

Change in fluorescence intensity in papillae, beneath the pollen grain, around the emerging pollen tube, or along the growing tube, was estimated by eye.

### Measurement of pollen hydration rate

Because the increase of water content in pollen grain is correlated with a change of its shape from ellipsoid to spheroid (Zuberi and Dickinson, 1985; Hiroi *et al.*, 2013; Wang *et al.*, 2017), for each pollen grain, we recorded every 2 min the ratio between the long and the wide axis (L/W) to estimate the pollen hydration rate until germination. Slopes were determined by linear regression of the hydration curve based on pollen ratio evolution during the first 10 min period (five data points), using the curve *f*=*ax*+*b*. The volume of the pollen grain was estimated using the formula for a prolate spheroid: 4/3×π×L/2×(W/2)^2^, where L=length of the longest axis and W=width of the pollen grain.

### Seed set

Flower buds at stage 12 were emasculated and pollinated with mature pollen 18 h later. At 21 d after pollination, mature siliques were opened to count the number of seeds.

### Statistical analysis

For each experiment (except the initial selection of SRK and SCR transgenic plants), at least two independent biological replicates (performed at different dates) were carried out. After checking that the mean and variability of the samples were homogenous between replicates, data were pooled to apply a statistical test. Graphs and statistics were obtained with R studio or Excel software. We tested the sample distribution with a Shapiro–Wilk test and applied a Student test to compare the mean of two given data sets. To compare multiple samples with a single test, we performed an ANOVA. In this study, *P*-values <0.05 were considered statistically significant.

## Results

### Generation of transgenic lines designed for live-cell imaging

We generated transgenic plants for *AlSRK14* in the Col-0 background as this accession is more suitable for crossing with available fluorescent marker lines. We used the *Brassica oleracea SLR1* (*S-locus-related gene 1*) promoter to express the *Al*SRK14 receptor in stigmatic cells. Among the 12 transgenic lines which we generated, we isolated four independent lines possessing a unique insertion and homozygous for *AlSRK14*. Stage 13–14E stigmas from these four lines rejected pollen from the original *A. lyrata S14* plant (less than five pollen tubes per stigma), whereas *A. lyrata* pollen produced numerous pollen tubes on Col-0 stigmas (Supplementary Fig. S1A–C). We quantified the expression level of the *AlSRK14* transgene in stage 13–14E stigmas of two of the strongest transgenic plants (lines #10 and #18) and found that the relative expression was twice as low as that detected in *A. lyrata S14* stigmas but still sufficient to induce the self-incompatibility response (Supplementary Fig. S1D). We selected the transgenic line #10 for further analysis and named it Col-0/*AlSRK14.* In spite of several attempts to introduce *AlSCR14* into Col-0, we did not succeed in generating transgenic pollen that could be rejected by stigmas of Col-0/*AlSRK14*. As an alternative, we used the C24 accession; this accession exhibits a strong self-incompatibility response when transformed with *A. lyrata SRK*/*SCR* genes (Nasrallah *et al.*, 2004; Liu *et al.*, 2007). We first transformed C24 plants with *AlSRK14* to generate a female tester capable of rejecting *A. lyrata S14* pollen grains (named C24/*AlSRK14* line #14; Supplementary Fig. S1C). Then, among the 18 transgenic lines transformed with *AlSCR14* in the C24 background, we isolated five independent lines with a unique insertion and homozygous for *AlSCR14*. Pollen from each of these lines was rejected on C24/*AlSRK14* line #14 mature stigmas (Supplementary Fig. S1E). We selected transgenic line #4 for further analysis, and it was named C24/*AlSCR14.*

**Fig. 1.**
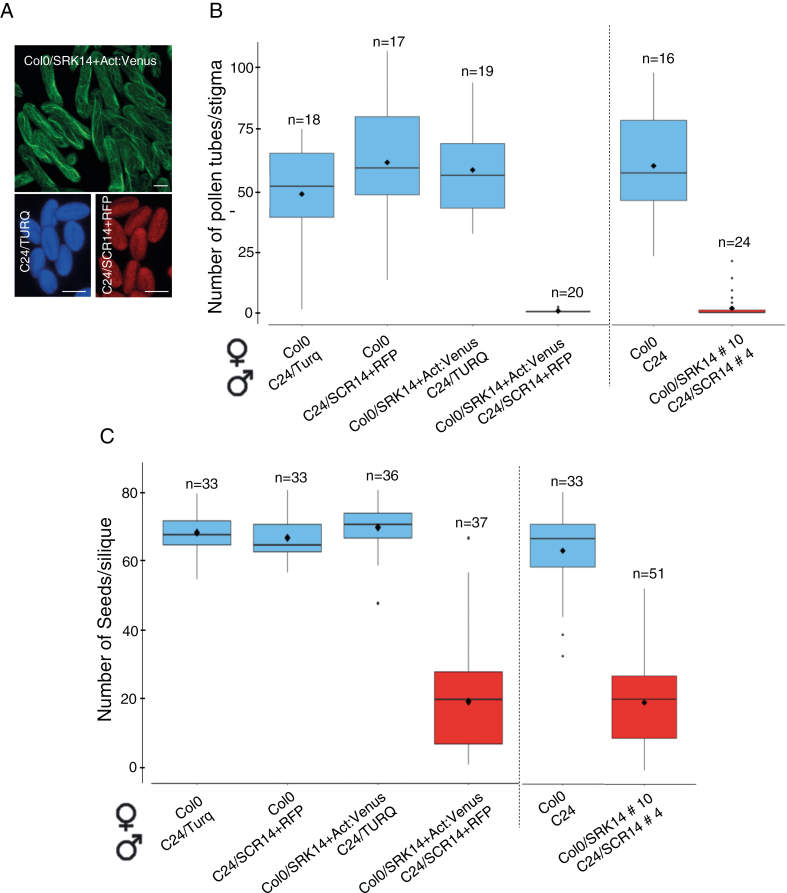
Generation of SI Arabidopsis lines. (A) The Col-0 transgenic line expressing *AlSRK14* was transformed with the fusion protein Act:Venus under the SLR1 promoter to label stigmas, whereas wild-type C24 or transgenic C24 expressing *AlSCR14* were transformed with the TURQUOISE protein (TURQ) or the RFP under pollen promoters, respectively. Scale bar=20 μm. (B) Self-incompatibility phenotype of generated lines. Stage 13–14E stigmas were pollinated with mature pollen, and pollen tubes present in stigmas were counted after aniline blue staining. In all crosses, the first name refers to the female partner and the second to the male partner (example: female Col0×male C24/Turq), (C) Seed set. Stage 13–14E stigmas were pollinated with mature pollen; after 21 d, siliques were dissected and seeds counted. *n*=number of pollinated stigmas. Mean (black diamond) of three independent experiments. Black dots are extreme values. Experiments separated by a dashed line were performed on different dates. Error bars indicate the SEM. (This figure is available in color at *JXB* online.)

To facilitate live-cell monitoring of pollen tube growth on the stigma, we introduced LifeAct fused to VENUS to label stigma papillae of Col-0/*AlSRK14* (Col-0/SRK14+Act:Venus), TURQ protein to monitor compatible pollen of C24 (C24/TURQ), and RFP for incompatible pollen of C24/*AlSCR14* (C24/SCR14+RFP) (Fig. 1A). We found that the presence of fluorescent proteins did not alter the compatibility (ANOVA test *P*=0.23) or the self-incompatibility response (*t*-test *P*-value=0.15) assessed by pollen tube counting (Fig. 1B). Despite the strong pollen rejection response following an incompatible cross (female Col-0/SRK14+Act:Venus×male C24/SCR14+RFP), some seeds developed in siliques (Fig. 1C). A comparable number of seeds was counted in siliques derived from an incompatible cross between non-labeled pollen and stigmas (mean values of 18.35 versus 19.04, *t*-test *P*=0.82, Fig. 1C). However, the incompatible crosses exhibited a significantly reduced seed set compared with the compatible crosses (*t*-test *P* =2.2×10^–16^ between the four compatible crosses cumulated and the two incompatible crosses cumulated, Fig. 1C). Together, our data show that we succeeded in restoring the self-incompatibility system in *A. thaliana* using a new *A. lyrata* haplotype *S14* and that a mature stigma recognizes and rejects incompatible pollen independently of the accession background. Futhermore, expressing a fluorescent protein in addition to the self-incompatibility determinant does not modify completion of the self-incompatibility response.

### Self-incompatibility response is maintained for about 2 d and then partially breaks down

**Fig. 2.**
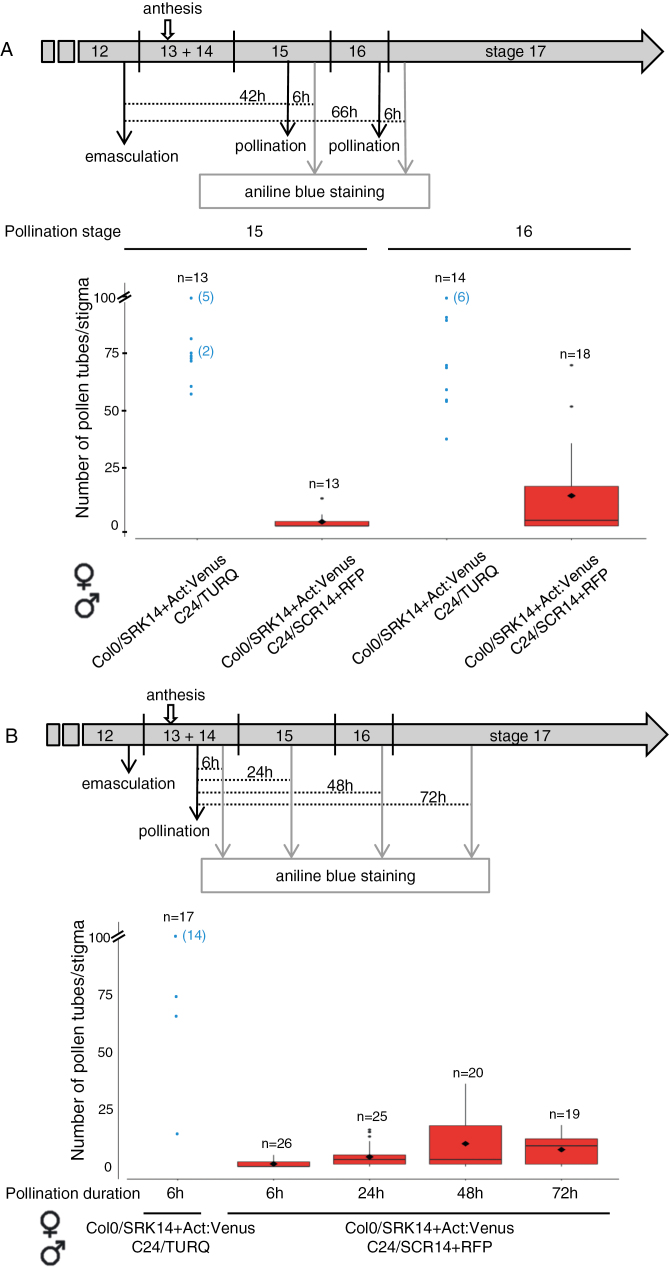
Dynamics of the self-incompatibility response in the Col-0 background. (A) Self-incompatibility phenotype following pollination of stigmas at two developmental stages. Flower buds at the end of developmental stage 12 were emasculated and pollinated with mature pollen 42 h (stigma stage 15) or 66 h (stigma stage 16) after emasculation (upper panel) and pollen tubes in stigmas were counted 6 h after pollination. Pollen tube count after aniline blue staining of stigmas (bottom graph). (B) Maintenance of the self-incompatibility reaction over time. Flower buds at the end of stage 12 were emasculated, pollinated with mature pollen 18 h later (stage13–14), and harvested 6, 24, 48, or 72 h after pollination (upper panel). Pollen tube count after aniline blue staining of stigmas (bottom graph). Beyond 100 pollen tubes per stigma, the exact number was not determined and was given a value of 100 (no mean, no error bar for compatible crosses). In all crosses, the first name refers to the female partner and the second to the male partner. *n*=number of pollinated stigmas. The gray dots correspond to individual pollinated stigma; when several stigmas have the same value, the stigma number is indicated in parentheses. Mean (black diamond) of two independent experiments. Black dots are extreme values. Error bars indicate the SEM. (This figure is available in color at *JXB* online.)

As some seeds could develop in siliques following incompatible crosses (Fig. 1C), we investigated how persistent the self-incompatibility response was during stigma development. To this end, we first carried out pollination experiments using compatible (C24/TURQ) or incompatible (C24/*AlSCR14*) pollen grains deposited on Col-0/SRK14+Act:Venus stigmas at different pistil developmental stages (Fig. 2A). When incompatible pollen grains were deposited on stage 15 stigmas, almost no pollen tube bypassed the stigmatic barrier (mean of 1.85 tubes per stigma, graph Fig. 2A), whereas some pollen tubes succeeded in penetrating stage 16 stigmas (mean of 13.2 tubes per stigma, graph Fig. 2A). In contrast, when compatible pollinations were performed on stigmas at stage 15 or 16, pollen tubes abundantly grew in the stigma. In a second set of experiments, we examined how long incompatible pollen grains deposited on stage 13–14E stigmas could stay inhibited after 6, 24, 48, or 72 h of contact with papilla cells (Fig. 2B). We observed a breakdown of the self-incompatibility barrier 48 h and 72 h after pollen deposition, when stigmas reached stage 16 and stage 17, respectively. However, the number of pollen tubes bypassing the self-incompatibility barrier (mean of 9.9 tubes per stigma and 7.3 tubes per stigma, respectively, graph Fig. 2B) was much less than the >50 tubes and stigma counted in compatible situations (graphs Fig. 2A, B). Together, these experiments show that a partial breakdown of the self-incompatibility response occurs late during pistil aging and that a strong self-incompatibility phenotype is maintained during a large time window of floral development, from stage 13 (anthesis) to stage 15 (stigma extended above long anthers), that is to say slightly less than 2 d (Smyth *et al.*, 1990). Thus the SI pollination partners we generated are suitable for developing live-cell imaging to monitor pollen behavior at the surface of mature stigmas (stage 13–14E) without risking weakening of the self-incompatibility barrier.

### Design of a semi-*in vivo* assay that allows live-cell imaging of early pollination events

**Fig. 3.**
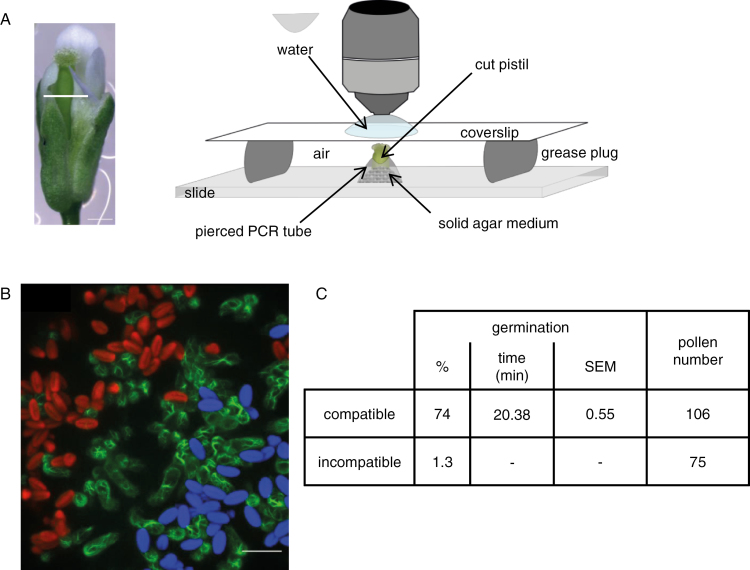
Set-up of the semi-*in vivo* assay. (A) The upper part of the pistil was cut (white line) and stuck in a solid agar medium. To control the humidity level, a small chamber was made with the bottom quarter of a PCR tube whose base was pierced with a needle and placed upside down on the medium. The stigma was pollinated and a cover slip was gently applied for live imaging. Scale bar=500 μm (B) Dual pollination on a single stigma. Stigma (Col-0/SRK14+Act:Venus) was pollinated first with incompatible pollen (C24/SCR14+RFP) and immediately after with compatible pollen (C24/TURQ), and pollen behavior was monitored under confocal microscopy for 32 min. The image is a *Z*-projection (2 min after pollen deposition). Scale bar=50 μm. (C) Pollen germination features extracted from five dual-pollinated stigmas. Only one incompatible grain germinated (germination time=28 min). (This figure is available in color at *JXB* online.)

To study the very early cellular changes that occur following pollen–stigma interaction, we designed a device that maintains the stigma alive for at least 1 h and allows live-cell imaging under confocal microscopy (Fig. 3A). In this system, compatible and incompatible pollen can be separately deposited on the same stigma and their behavior tracked in the same experimental conditions (Fig. 3B, Supplementary Video S1). To test whether this set-up permitted the maintenance of compatibility/incompatibility responses, we performed five independent experiments where a Col-0/SRK14+Act:Venus stigma was dual pollinated with C24/SCR14+RFP (incompatible) and C24/TURQ (compatible) pollen grains. We found that the percentage of germination for compatible pollen varied from 58% to 81%, with a mean value of 74% (*n*=106, Fig. 3C). Emission of a pollen tube occurred on average 20 min after pollen deposition (Fig. 3C). On the contrary, germination of incompatible pollen was a rare event, occurring for only 1.3% of the tracked grains (*n*=75, Fig. 3C). Thus, our semi-*in vivo* system allows live-cell imaging of pollinated stigmas while preserving the highly regulated self-incompatibility process.

### Actin dynamics in stigmatic cells during pollination

**Fig. 4.**
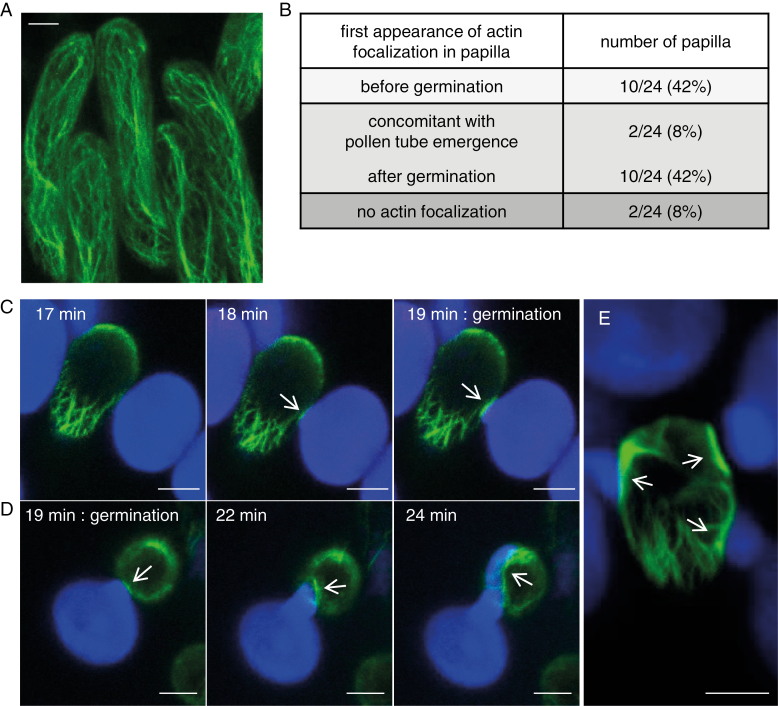
Actin reorganization at the contact site with the compatible pollen grain and pollen tube. (A) Visualization of actin bundles in unpollinated stigmatic cells expressing the Act:Venus marker (Col-0/SRK14+Act:Venus). The image is a *Z*-projection. (B) Dynamics of actin focalization following compatible pollination. Act:Venus stigmas were dual pollinated in the semi-*in vivo* system and a *Z*-stack was taken every minute during 32 min. Twenty-four papillae in contact with a compatible pollen grain were selected on 14 stigmas and the first appearance of actin fluorescence (focalization) was recovered. Timing of actin focalization was defined regarding the pollen development stage. (C) Actin reorganization in papillae just before pollen germination. (D) Actin reorganization in papillae during germination and pollen tube growth. The indicated time corresponds to time after pollen deposition. Images are *Z*-projections. (E) Mutiple pollen germination on a single papilla. The image is a *Z*-projection. Arrows show the focalization of actin fluorescence. Scale bar=10 μm. (This figure is available in color at *JXB* online.)

Because remodeling of the actin cytoskeleton in the papilla cells has been reported following pollination in Brassica (Iwano *et al.*, 2007), we decided to monitor actin dynamics over time. Before pollination, we found the actin cytoskeleton network to be composed of bundles mainly oriented along the longitudinal axis of the stigmatic cell (Fig. 4A). Upon compatible pollination, a clear accumulation of actin that focalized at the contact site with the compatible pollen was observed in 92% of the pollinated papillae (22/24, Fig. 4B). For 42% of the stigmatic cells (10/24), actin fluorescence increased beneath the pollen grain just before germination. For 50% of the cells, actin focalized when a clear emergence of the tube (minimum 1 μm) was visible (8%, 2/24) or when the tube started to grow on the stigma surface (42%, 10/24) (Fig. 4B, C; Supplementary Video S2; Supplementary Fig. S3). At later stages of pollen tube progression, actin focalization was observed all along the tube path in 83% (20/24) of the monitored papilla cells (Fig. 4D; Supplementary Video S3). Interestingly, when several pollen germinated on the same papilla, actin focalization was observed below each tube (Fig. 4E). In contrast, on the seven dual-pollinated stigmas we tracked over time, we never detected any actin focalization beneath the incompatible pollen grains (Supplementary Video S4). All together, our live-cell imaging analysis shows that the actin cytoskeleton polymerizes in the stigmatic cell following deposition of compatible but not incompatible pollen grains. Actin accumulation at the pollen contact occurs during the germination process and goes on throughout the elongation of the pollen tube.

### Kinetics of compatible pollen hydration is divided into two phases

**Fig. 5.**
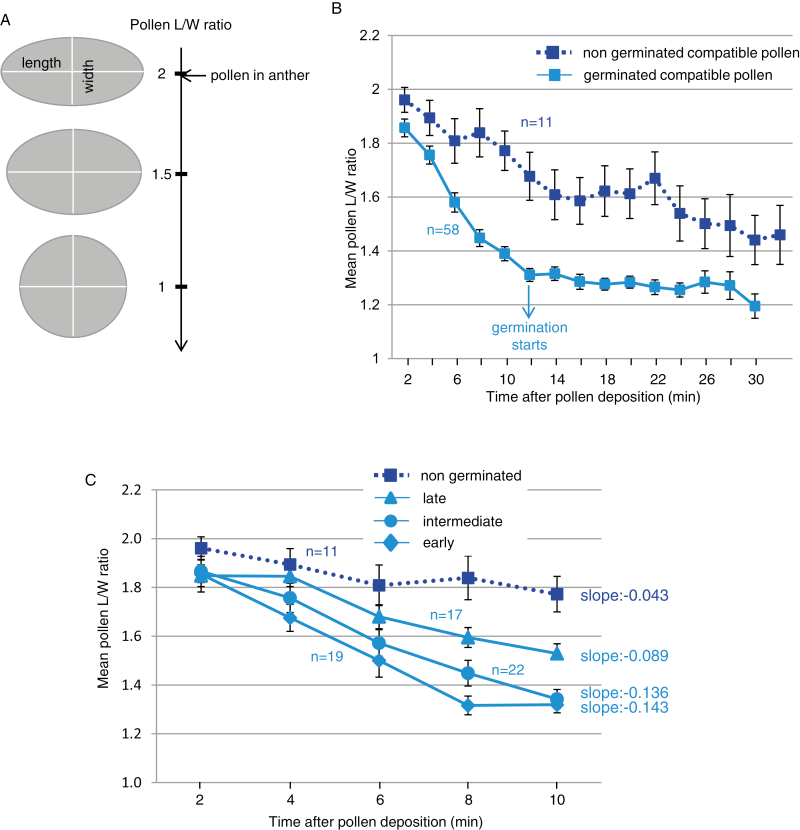
Kinetics of compatible pollen hydration following dual pollination. (A) During hydration, pollen shape changes from an ovoid towards a round shape given by the length/width ratio (L/W ratio). (B) Evolution of the L/W ratio during a compatible interaction. Among the 69 tracked pollen grains, 58 germinated during the experiment (solid line), whereas 11 failed to germinate (dashed line). (C) Kinetics of pollen hydration related to rate of germination. Pollen grains were classified by their germination time in four categories: early, intermediate, late, and non-germinated. Slopes were calculated by linear regression based on the pollen hydration curve during the first 10 min. *n*=number of pollen grains. Mean of five independent experiments. Error bars indicate the SEM. (This figure is available in color at *JXB* online.)

Next, we compared the kinetics of pollen hydration and germination following dual pollination, using the L/W ratio to estimate the pollen hydration level (Zuberi and Dickinson, 1985). At maturity, the mean L/W ratio of pollen grains was close to two (Fig. 5A; Supplementary Table S2). We measured the pollen ratio every 2 min, starting 2 min after pollen deposition (Fig. 5B; Supplementary Fig. S2). Among the 69 compatible pollen grains we tracked, 58 germinated in the course of the experiment whereas 11 never did. We found that during the first 10 min (preceding germination, Supplementary Table S3), the mean pollen ratio dramatically decreased, indicating that grains became rounded (solid curve Fig. 5B). Interestingly, over the same time period, the pollen volume increased (3455 μm^3^ at 2 min and 4974 μm^3^ at 10 min), strongly supporting that pollen swelling is linked to water uptake and strengthening the use of the L/W ratio as a proxy for pollen hydration. During the following 20 min, water uptake almost stopped; indeed, there was no significant difference between the 12 min and 30 min L/W ratios (*t*-test *P*=0.71). We found that germination started when the pollen ratio was <1.4 (solid curve Fig. 5B). Among the 58 pollen grains that germinated during the course of the experiment, a huge majority (57/58) reached the 1.4 ratio before germination; only one germinated pollen grain never reached this ratio. For the 11 compatible pollen grains that never germinated (dashed curve Fig. 5B), the hydration curve shows no rapid hydration phase during the first 10 min and the mean ratio never reached the value of 1.4. Taken together, our results show that in Arabidopsis, water transport toward the pollen grain follows biphasic kinetics: first, an initial phase characterized by a high pollen hydration rate required to reach a certain degree of water content in the pollen (ratio 1.4), followed by a second phase where the hydration rate dramatically decreases and pollen germination occurs.

### Variability in pollen germination time depends on the hydration rate during the early interaction phase

In our experimental conditions, pollen germination was not synchronous, starting 12 min after pollen contact with the stigma and spreading over the 32 min of the experiment (Supplementary Table S3). We suspected that different hydration rates of pollen grains might be the cause of this variability. To test this hypothesis, we classified the 69 compatible pollen grains according to their germination capability and categorized them into four classes: those that germinated early (from 12 min to 18 min, *n*=19); intermediate (from 20 min to 24 min, *n*=22); late (from 26 min to 32 min, *n*=17), and those that did not germinate (*n*=11), and we compared their hydration rate during the rapid hydration phase from 2 min to 10 min (Fig. 5C). At 2 min, no significant differences were found between the L/W ratios of pollen for each classes (1.85/1.87/1.85/1.96, respectively; ANOVA test *P* =0.13, Fig. 5C). At 10 min, pollen that germinated late were significantly less hydrated (higher L/W ratio) than those that germinated early or were intermediate (*t*-test *P* =2.43×10^–4^ and 1.64×10^–3^, respectively, Fig. 5C), though their hydration degree was significantly higher than that of non-germinated pollen (*t*-test *P* =3.62×10^–2^, Fig. 5C). We calculated the slopes of the hydration curves by linear regression, as previously described (Wang *et al.*, 2017), and found that early and intermediate germinating pollen grains had the highest hydration rates compared with late and non-germinating pollen grains (Fig 5C). Non germinating pollen exhibited a very slow hydration rate (slope –0.043) associated with a poor water uptake during the first 10 min, deduced from the high pollen L/W ratio at 10 min (mean of 1.77). Our data show that most of the tracked compatible pollen germinated within 32 min and that the germination time relates to the capacity of the pollen to take up water during an initial short period of 10 min. The faster the pollen takes water from the stigma, the quicker it germinates.

### Incompatible pollen grains do not fully hydrate and pollen tubes do not penetrate the stigmatic cell wall

**Fig. 6.**
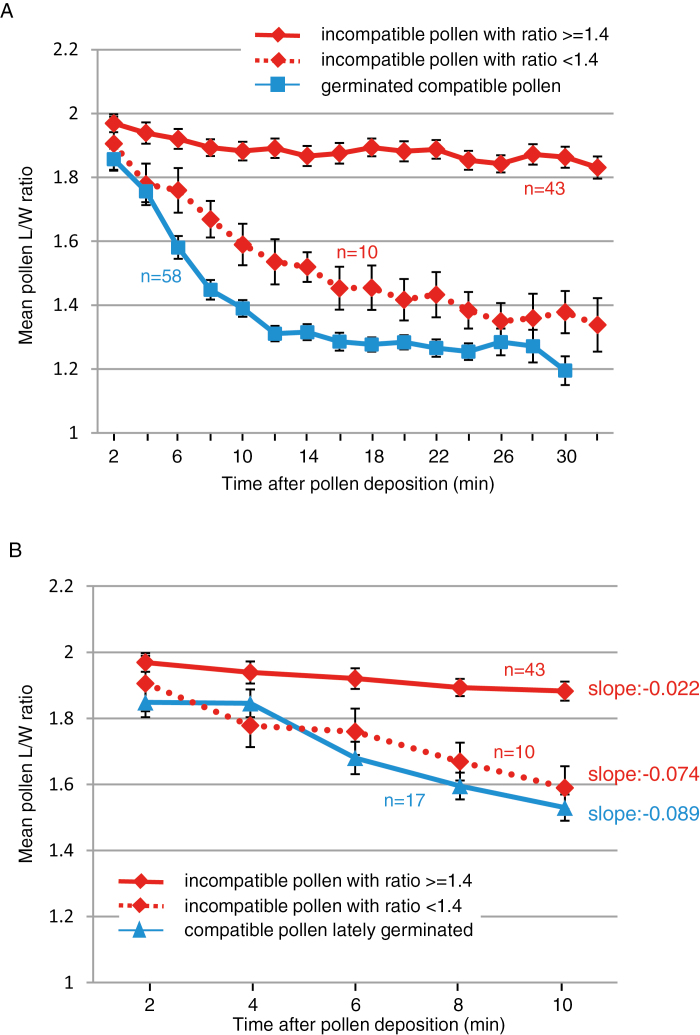
Kinetics of incompatible pollen hydration following dual pollination. (A) Evolution of the L/W ratio during an incompatible interaction. Among the 53 tracked pollen grains, 43 exhibited an L/W ratio that never reached a ratio below 1.4, whereas 10 had a ratio below 1.4 (dashed red line). The solid line that indicates hydration dynamics of compatibe pollen was reproduced from Fig. 5B to facilitate the comparison. (B) Hydration rates during the first 10 min of incompatible pollen with an L/W ratio ≥1.4, incompatible pollen with an L/W ratio <1.4, and compatible pollen that germinated late (solid line, reproduced from Fig. 5C). Slopes were calculated by linear regression. *n*=number of pollen grains. Mean of five independent experiments, error bars indicate the SEM. (This figure is available in color at *JXB* online.)

Next, we examined the hydration rate of incompatible pollen on the dual-pollinated stigmas. Of the 53 incompatible grains we monitored, none germinated during the time course of the experiment. Futhermore, most of them (*n*=43) never exhibited an L/W ratio below the threshold value of 1.4 (Fig. 6A) and only hydrated poorly compared with compatible grains (Fig. 6A). The mean hydration curve for these 43 incompatible grains showed a constant slope during the 32 min of the experiment. In contrast, the 10 remaining incompatible grains reached an L/W ratio below 1.4 with a mean hydration curve (Fig. 6A) resembling that of compatible pollen. In addition, their hydration rate within the first 10 min was similar to that of late germinating compatible pollen (slope value of –0.074 versus –0.089, respectively, Fig. 6B), and hence hydrated as efficiently as some compatible grains.

**Fig. 7.**
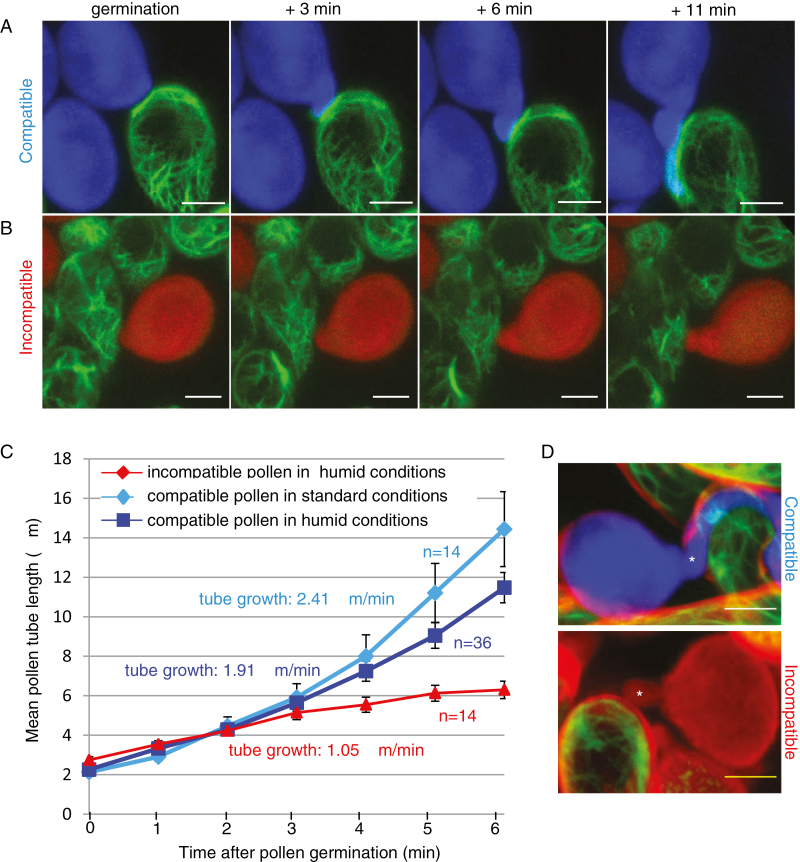
Pollen germination in high humidity conditions. (A) Compatible pollen was deposited on an Act:Venus stigma and incubated in high humidity conditions. (B) Incompatible pollen was deposited on an Act:Venus stigma and incubated in high humidity conditions. A *Z*-stack was taken every minute after pollen deposition. Images are Z-projections. Scale bar=10 μm. (C) Growth rate of pollen tubes in standard and high humidity conditions. Pollen tube length was measured at each time point from germination (0 on the horizontal axis) during 6 min, for compatible pollen in standard conditions, in high humidity conditions, or for incompatible pollen in high humidity conditions. *n*=number of tracked pollen grains on a minimum of six different stigmas; error bars indicate the SEM. (D) At 35 min after incubation in high humidity conditions, stigmas were stained with FM4-64. A *Z*-stack was processed with ImageJ to generate a *Z*-projection. A compatible pollen tube (asterisk, upper panel) was detected beneath the FM4-64-labeled layer, whereas an incompatible tube (asterisk, lower panrl) was outside. Scale bar=10 μm. (This figure is available in color at *JXB* online.)

As high humidity can stimulate pollen hydration and promote incompatible pollen germination (Carter and McNeilly, 1976; Ockendon, 1978; Zuberi and Dickinson, 1985), we checked the effect of high humidity (100%) on pollen behavior in a modified semi-*in vivo* system (Supplementary Fig. S3). We found that compatible pollen germination was slightly stimulated compared with standard conditions (88% germination versus 74% and mean germination time of 18.55 min versus 20.38 min, respectively; Supplementary Fig. S5). Moreover, high humidity conditions did not dramatically affect actin reorganization as we detected a clear focalization of actin beneath the pollen grain/tube in 75% of the pollinated papillae (33/44) (Fig. 7A; Supplementary Fig. S5; Supplementary Video S5). Following incompatible pollination at high humidity, we observed that germination of incompatible pollen was strongly stimulated, varying from 18% to 58% depending on the experiment (mean of 35%, Supplementary Fig. S6) compared with the 1.3% of germination in standard conditions. In addition, among the 46 grains monitored, 46% reached an L/W ratio of 1.4 within 10 min (Supplementary Table S4), whereas only 4% reached this value in standard conditions. However, it took more time for incompatible pollen tubes to emerge compared with compatible tubes (24.87 min versus 18.55 min, Supplementary Fig. S6), and the tube seemed blocked at the papilla surface, the pollen being detached from the papilla (Fig. 7B; Supplementary Video S6). The growth rate of an incompatible tube was 1.05 μm min^–1^, whereas a compatible tube extended much more rapidly in both standard and high humidity conditions (2.41 μm min^–1^ and 1.91 μm min^–1^, respectively, Fig. 7C). Growth kinetics show that during the first 3 min, the elongation rate of both compatible and incompatible tubes was comparable, but then compatible tubes extended rapidly whereas incompatible tubes slowed down (Fig. 7C). These observations suggest that the incompatible tube may not be able to penetrate the papilla cell wall. To test this hypothesis, we stained papilla cells with the amphiphilic styryl dye FM4-64. In contrast to most plant cells where the dye labels the plasma membrane and the vesicular network (Grebe *et al.*, 2003; Jaillais *et al.*, 2008; Jelínková *et al.*, 2010), we noticed that it remained at the surface of the papilla as it did not co-localize with the actin or the plasma membrane canonical marker LTI6b (Supplementary Fig. S6). This labeling is probably due to the interaction of the dye with the hydrophobic cuticle layer of the stigmatic cell. While the compatible pollen tube clearly penetrated the FM4-64-labeled layer and grew in close contact with the papilla cytoplasm identified by the actin fluorescence (9 tubes/9, Fig. 7D), the incompatible tube was never observed under the FM4-64 layer and stayed outside the papilla (11 tubes/11, Fig. 7D). Additionally, at the contact site with the short abnormal incompatible tube, we rarely observed focalization of actin in the papilla, contrary to the compatible situation (Fig. 7B; Supplementary Fig. S5; Supplementary Video S6). Taken together, our results show that incompatible grains generally fail to fully hydrate and, when pollen hydration and germination are promoted by high relative humidity, the self-incompatibility reaction efficiently blocks tube penetration into the papilla cell wall, the tube remaining outside the papilla surface without triggering actin focalization at the contact site with the stigmatic cell.

## Discussion

Here, we set up a live imaging system with a fast-scanning confocal microscope to simultaneously monitor compatible and incompatible Arabidopsis pollen behavior on the same single stigma and provide a thorough analysis of the dynamics of the cellular events following pollen perception.

### *AlSCR14* and *AlSRK14* SI Arabidopsis lines

The two Arabidopsis lines we generated, one that expressed the female determinant (*Al*SRK14) and the second the male determinant (*Al*SCR14), were self-fertile but exhibited a strong self-incompatibility response when crossed. This self-incompatibility response was maintained during flower development for ~2 d, only weakening at late stages of stigma development where few pollen tubes grew in pistil tissues and ultimately led to seed set. Nevertheless, the seed yield remained much below the one reported for selfed Col-0 Arabidopsis (19 seeds per silique versus 62 seeds per silique). Thus, these SRK14 and SCR14 transformants almost fully recapitulate the self-incompatibility phenotype of the natural SI Brassicaceae, the strength of the response, however, being less persistent compared with strictly SI lines. Previous work reported a strong self-incompatibility phenotype, although over a narrow window, in stigmas at stage 13 and early stage 14 in Col-0 lines expressing the cognate *A. lyrata SCRb–SRKb* gene pair (the so-called Col-0/*AlSb* line), whereas older flowers fully accepted self-pollen (Nasrallah *et al.*, 2002; Nasrallah *et al.*, 2004). In contrast, in another study, the same *SCRb–SRKb* gene pair was shown to be unable to confer self-incompatibility in Col-0, even in stage 13 flowers, the transgenic plants being fully fertile with a seed set similar to selfed Col-0 plants (Indriolo *et al.*, 2014). In this latter study, a role for the E3-ubiquitin ligase ARC1 in promoting self-incompatibility was demonstrated as only Col-0 co-expressing the *A. lyrata* or *B. oleracea* ARC1 with the *SCRb–SRKb* pair acquired the capacity to reject self-pollen. Interestingly, the Col-0/*AlSRK14* lines generated in our study express a strong self-incompatibility response in mature stigmas without the need for co-expressing *AlARC1*, the stigmas remaining partially self-incompatible at later flower developmental stages. Earlier work showed that the variability of self-incompatibility phenotypes observed between Arabidopsis transformants was mainly due to *SRK* transcript levels (Nasrallah and Nasrallah, 2014). It has been reported that the transient self-incompatibility phenotype and self-fertility of Col-0/*Al*S*b* transformants were caused by modifier loci harbored in the Col-0 genome (Liu *et al.*, 2007; Boggs *et al.*, 2009). One of these loci, *PUB8*, encoding an ARM-repeat and U-box protein, regulates *SRK* transcript levels and is responsible for the pseudo-self-compatibility observed in old flowers of Col-0/*AlSb* (Liu *et al.*, 2007). In contrast to the Col-0/*AlSb* transformants mentioned above, in our study, *AlSRK14* expression was driven by the *SLR1* promoter region that does not contain the *SRK* 5' and 3' regulatory sequences. As a consequence, it is likely that transcription of the *AlSRK14* gene and/or stability of the transcripts escape the regulation of Col-0 self-incompatibility modifiers, leading to the accumulation of AlSRK14 protein to a level sufficient to maintain a partial pollen rejection response during flower development. The weakening of the self-incompatibility response we observed in late developmental stages was expected as it is known that *SLR1* promoter activity is developmentally regulated, decreasing with flower aging (Lalonde *et al.*, 1989), and hence leading to lower abundance of *SRK14* transcript levels.

Though we attempted to transform Col-0 with *AlSCR14*, we failed to obtain transformants whose pollen grains were rejected on *AlSRK14*-expressing stigmas. In SI Arabidopsis species, a dominance hierarchy between *SCR* alleles has been reported, which is regulated by small RNAs (sRNAs) (Durand *et al.*, 2014). We may propose that the inability to introduce a functional *AlSCR14* in Col-0 might reside in the presence of vestigial sRNAs targeting the recessive *AlSCR14* allele, while these sRNAs would be absent in the C24 background.

### Pollen behavior

We observed that ~20 min after interaction with the stigma papillae, the compatible pollen germinates a tube, which is consistent with the germination time reported in Arabidopsis after *in vivo* pollination (Kandasamy *et al.*, 1994; Iwano, 2004). Once emerged, the pollen tube extends at a rate of 2.41 μm min^–1^, which is significantly faster than tube elongation *in vitro* (~1 μm min^–1^; Iwano *et al.*, 2004; Yang *et al.*, 2017) and consistent with growth rates reported in other *in vivo* systems (Iwano, 2004; Cheung *et al.*, 2010). Futhermore, we found that the tube growth rate is not constant, exhibiting a slow growth phase within the first 3 min after tube emergence, followed by a second phase of more rapid growth. Biphasic growth kinetics were already reported for Arabidopsis pollen tubes growing *in vitro*, except that the timing was much longer; the transition from germination to a rapid growth phase required 30 min (Vogler *et al.*, 2015). While compatible pollen grains promptly achieve germination, incompatible grains deposited on the same stigma, and in the close vicinity of compatible ones, have a completely different destiny. Indeed, incompatible pollen grains exhibit an extremely low germination rate (1.3%), which is in agreement with the few pollen tubes we detected after aniline blue staining. Thus, our semi-*in vivo* system recapitulates what happens in nature when pollen from various origins lands on the same stigma, accepting compatible but rejecting incompatible pollen grains.

We found that almost immediately after landing on the stigma, compatible pollen starts to hydrate and within 10 min is almost fully hydrated. In contrast, during the same time period, the L/W ratio of incompatible grains only poorly evolves. Thus, inhibition of the incompatible pollen acts very early, within the first minutes following stigma contact, blocking pollen hydration. As previously described, hydration appears as the first check point controlling pollen rejection in SI species with dry stigmas (Zuberi and Dickinson, 1985; Dickinson, 1995; Samuel *et al.*, 2009; Hiroi *et al.*, 2013; Safavian and Goring, 2013; Wang *et al.*, 2017). In this study, the hydration threshold required to trigger germination was found to correspond to a pollen L/W ratio of <1.4. Pollen turgor pressure has been proposed to be the main driving force for germination (Hiroi *et al.*, 2013; Vogler *et al.*, 2015; Wang *et al.*, 2017). Our result suggests that the optimal pollen turgor pressure corresponds to a 1.4 L/W ratio which is reached after 10 min of pollen deposition.

Although 19% (10/53) of the incompatible grains exhibit hydration features similar to compatible grains, they, however, never germinate. Thus, a second check point acts to ensure that undesirable pollen grains that escape the hydration control are properly inhibited, blocking their germination. When hydration of incompatible pollen is artificially boosted in a saturated humidity environment, a significant proportion of the grains bypass the second checkpoint and germinate. However, pollen tube growth is slow and does not reach the second phase of rapid elongation observed with compatible tubes. Futhermore, tubes grow outside the papilla cell, unable to penetrate the cell wall. Production of a short incompatible tube, whose growth is arrested in papilla cells, has been described in some *S*-haplotypes of SI Brassica species (Elleman and Dickinson, 1994; Dickinson, 1995). Thus, our data highlight this late control of the self-incompatibility reaction that prevents penetration of the stigmatic cell wall by incompatible tubes.

The current self-incompatibility model in the Brassicaceae proposes that the signaling cascade triggered by SRK activation disrupts the delivery of stigmatic secretory vesicles required for compatible pollen acceptance (reviewed in Jany *et al.*, 2019). The content of these vesicles is currently unknown but, regarding the three inhibition levels described in this study and others (Zuberi and Dickinson, 1985; Haasen *et al.*, 2010), we may postulate that stigmatic vesicles transport mainly water to hydrate the pollen grain and trigger germination as well as enzymatic activities to prepare the stigmatic cell wall for pollen tube penetration.

### Actin cytoskeleton

In Brassica, remodeling of the actin cytoskeleton architecture has been described at the site of pollen–stigma interaction (Iwano *et al.*, 2007). However, these results remain controversial, as a previous work suggested that actin filaments do not show any rearrangement (Dearnaley *et al.*, 1999). Using an actin marker line, we show that a clear reorganization of actin occurs at the contact site with compatible pollen. However, while actin bundles appear in Brassica papillae as soon as pollen hydration starts and remain visible during the entire pollen hydration process (Iwano *et al.*, 2007), in Arabidopsis, actin reorganization starts later, from the end of pollen hydration (just before germination) or following pollen tube emergence. It was previously proposed that the self-incompatibility mechanism in *Brassica* and Arabidopsis does not rely on rigorously similar cellular processes (Kitashiba *et al.*, 2011). Alternatively, these discrepancies could be due to technical reasons: (i) we used stable transformed Arabidopis lines instead of transient expression in *Brassica* papillae; (ii) we performed massive pollination of the stigmatic cells compared with deposition of a single *Brassica* pollen by micromanipulation; and (iii) we used the LifeAct peptide instead of the mouse Talin protein to label F-actin because this short peptide does not affect actin dynamics *in vivo* (Era *et al.*, 2009). Nevertheless, whatever the species, actin focalization appears as a hallmark of the compatible reaction as it is never detected in incompatible pollination. Interestingly, Hardham *et al.* (2008) showed that touching the surface of the *A. thaliana* cotyledon epidermal cells with a microneedle induced a rapid actin focalization around the contact point, leading the authors to propose that actin reorganization is triggered by detection of mechanical pressure. Likewise, reorganization of actin microfilaments was observed in many plant–pathogen interactions, where actin cables accumulate at the contact site with the pathogen (Takemoto and Hardham, 2004, and references therein). Altogether, we may hypothetize that reorganization of actin is triggered in stigmatic cells that sense the mechanical pressure produced by the pollen grain/tube at the site of cell wall penetration. In accordance with this, we generally did not detect modification of the actin architecture at the contact site with incompatible pollen tubes that were generated in high humidity conditions and that could not penetrate the stigmatic cell wall. Alternatively, the pollen grain, when engaged in compatibility, could also initiate a signaling cascade leading to the actin polymerization at the contact site with the germinating grain and/or the growing pollen tube. Nevertheless, the focalization of actin is consistent with the well-characterized role of the actin network in vesicular delivery to the plasma membrane (Szymanski and Staiger, 2018) induced upon compatible pollination.

Taken together, using fast-scanning confocal microscopy, our observations underline the suitability of our system to capture very dynamic events occurring within minutes. Notably, we identified a hydration threshold (L/W <1.4) that pollen grains must reach to acquire the critical water content required for supporting pollen germination. In addition, we monitored the dynamics of the actin network during compatible and incompatible responses and found that focalization of actin cables was triggered when pollen–stigma interaction is engaged in compatibility. This actin reorganization might be elicited in response to the mechanical pressure exerted by pollen grain/tube or alternatively by a specific factor delivered by the pollen. Finally, our semi-*in vivo* system provides a particularly well-suited system to test putative components involved in the acceptance or rejection of pollen grains through the analysis of Arabidopsis mutants as well as transgenic lines, and will make possible the deciphering of the early events controlling sexual reproduction.

## Supplementary data

Supplementary data are available at *JXB* online.

Table S1. Primers used in this study.

Table S2. L/W ratio of pollen grains coming out of mature anthers.

Table S3. Germination of compatible pollen tracked during experiments described in Fig. 5.

Table S4. L/W ratio of incompatible pollen in high humidity conditions 10 min after pollen deposition.

Fig. S1. *AlSRK14* and *AlSCR14* transgenic plant selection.

Fig. S2. Dynamics of actin focalization following compatible pollination.

Fig. S3. Hydration kinetics of compatible and incompatible pollen in the semi-*in vivo* system.

Fig. S4. Standard and high humidity assays.

Fig. S5. Behavior of compatible pollen in high humidity conditions.

Fig. S6. Behavior of incompatible pollen in high humidity conditions.

Fig. S7. FM4-64 labeling of stigmatic cells.

Video S1. Dual pollination with compatible and incompatible pollen deposited on the same stigma.

Video S2. Actin rearrangement at the pollen contact site.

Video S3. Actin rearrangement along the pollen tube path.

Video S4. Actin behavior in stigmatic cells in contact with incompatible pollen.

Video S5. Compatible pollen germination and pollen tube growth in high humidity conditions.

Video S6. Incompatible pollen germination and pollen tube growth in high humidity conditions.

eraa008_suppl_supplementary_video_S1

eraa008_suppl_supplementary_video_S2

eraa008_suppl_supplementary_video_S3

eraa008_suppl_supplementary_video_S4

eraa008_suppl_supplementary_video_S5

eraa008_suppl_supplementary_video_S6

eraa008_suppl_supplementary_video_legends

eraa008_suppl_supplementary_figures_S1_S7_tables_S1_S4
