# Supplementary material for: Live-cell imaging of early events following pollen perception in self-incompatible Arabidopsis thaliana
Source: J Exp Bot. 2020 Jan 14;71(9):2513–26. doi: 10.1093/jxb/eraa008 (PMC7210763; doi:10.1093/jxb/eraa008)
Supplement: eraa008_suppl_supplementary_video_legends [file eraa008_suppl_supplementary_video_legends.pdf]

Supplementary Data

Title: Live-cell imaging of early events following pollen perception in self-incompatible *Arabidopsis thaliana*

Authors: Frédérique Rozier, Lucie Riglet, Chie Kodera, Vincent Bayle, Eléonore Durand, Jonathan Schnabel, Thierry Gaude and Isabelle Fobis-Loisy

Video S1: Dual pollination with compatible and incompatible pollen deposited on the same stigma

Video S2: Actin rearrangement at the pollen contact site

Video S3: Actin rearrangement along the pollen tube path

Video S4: Actin behavior in stigmatic cells in contact with incompatible pollen

Video S5: Compatible pollen germination and pollen tube growth in high humidity conditions

Video S6: Incompatible pollen germination and pollen tube growth in high humidity conditions

Supplementary video S1

Supplementary video S1. Dual pollination with compatible and incompatible pollen deposited on the same stigma. A stigma expressing the Act:Venus marker (green fluorescence) was pollinated first with incompatible pollen (left part of the stigma, red fluorescence) and immediately after with compatible pollen (right part of the stigma, blue fluorescence). A Z-stack was taken every two minutes after pollen deposition. Images were processed with ImageJ to generate a Z-projection at each time point to construct the video. Indicated time corresponds to time after pollen deposition. Bar = 50 μm.

Supplementary video S2

Supplementary video S2. Actin rearrangement at the pollen contact site. An Act:Venus stigma (green fluorescence) was dual-pollinated and we focused on one stigmatic cell in contact with a compatible pollen (blue fluorescence). A Z-stack was taken every minute after pollen deposition. Images were processed with Image J to generate a Z-projection at each time point to construct the video. Indicated time corresponds to time after pollen deposition. Bar = 10 μm.

Supplementary video S3

Supplementary video S3. Actin rearrangement along the pollen tube path. An Act:Venus stigma (green fluorescence) was dual-pollinated and we focused on one stigmatic cell in contact with a compatible pollen (blue fluorescence). A Z-stack was taken every minute after pollen deposition. Images were processed with image J to generate a Z-projection at each time point to construct the video. Indicated time corresponds to time after pollen deposition. Bar = 10 μm.

Supplementary video S4

Supplementary video S4. Actin behavior in stigmatic cells in contact with incompatible pollen. An Act:Venus stigma (green fluorescence) was dual-pollinated. A Z-stack was taken every minute after pollen deposition. Images were processed with image J to generate a Z-projection at each time point to construct the video. Indicated time corresponds to time after pollen deposition. The selected stigma is representative of the seven tracked stigmas. Bar = 10 μm.

Supplementary video S5

Supplementary video S5. Compatible pollen germination and pollen tube growth in high humidity conditions. An Act:Venus stigma (green fluorescence) was pollinated with compatible pollen (blue fluorescence) and incubated in high humidity conditions. A Z-stack was taken every minute after pollen deposition. Images were processed with image J to generate a Z-projection at each time point to construct the video. Indicated time corresponds to time after pollen deposition. Video starts from pollen germination. Bar = 10 μm.

Supplementary video S6

Supplementary video S6. Incompatible pollen germination and pollen tube growth in high humidity conditions. An Act:Venus stigma (green fluorescence) was pollinated with incompatible pollen (red fluorescence) and incubated in high humidity conditions. A Z-stack was taken every minute after pollen deposition. Images were processed with image J to generate a Z-projection at each time point to construct the video. Indicated time corresponds to time after pollen deposition. Video starts from pollen germination. Bar = 10 μm.
