# Supplementary material for: Live-cell imaging of early events following pollen perception in self-incompatible Arabidopsis thaliana
Source: J Exp Bot. 2020 Jan 14;71(9):2513–26. doi: 10.1093/jxb/eraa008 (PMC7210763; doi:10.1093/jxb/eraa008)

Supplementary Data

Title: Live-cell imaging of early events following pollen perception in self-incompatible *Arabidopsis thaliana*

Authors: Frédérique Rozier, Lucie Riglet, Chie Kodera, Vincent Bayle, Eléonore Durand, Jonathan Schnabel, Thierry Gaude and Isabelle Fobis-Loisy

Table S1: Primers used in this study

Figure S1: *AlSRK14* and *AlSCR14* Transgenic plant selection

Table S2: L/W ratio of pollen grains coming out of mature anthers

Figure S2: Dynamics of actin focalization following compatible pollination

Figure S3: Hydration kinetics of compatible and incompatible pollens in the semi *in vivo* system

Table S3: Germination of compatible pollen tracked during experiments described in figure 5

Figure S4: Standard and high humidity assays

Figure S5: Behavior of compatible pollen in high humidity conditions

Figure S6: Behavior of incompatible pollen in high humidity conditions

Table S4: L/W ratio of incompatible pollen in high humidity conditions 10 minutes after pollen deposition

Figure S7: FM4-64 labelling of stigmatic cells

Supplementary table S1

Supplementary table S1. Primers used in this study

name	Sequence 5'-- 3'	use
AlSRK14-AttB1	GGGGACAAGTTTGTACAAAAAAGCAGGCT ACCATGAGAGGTGTAATACCAAAGTACC	*AlSRK14* gene cloning
AlSRK14-AttB2	GGGGACCACTTTGTACAAGAAAGCTGGGT TTACCGAGGTTCCACTTCCGTGGTGG	*AlSRK14* gene cloning
AlSCR14-AttB1	GGGGACAAGTTTGTACAAAAAAGCAGGCT CGGGTAGCTCAACCTAGCTAAG	*AlSCR14* cloning (including promoter, gene and 3'UTR)
AlSCR14-AttB2	ACCACTTTGTACAAGAAAGCTGGGTCATG ATCACCAAAGACAAGATCC	*AlSCR14* cloning (including promoter, gene and 3'UTR)
Actin8-F	CGACGGACAAGTGATCACGATC	RT-PCR, *Actin8* amplification
Actin8-R	CATAGTTGTACCACCACTGAGCAC	RT-PCR, *Actin8* amplification
AlSRK14-F	GCCGCCAGACACATCCGGGGC	RT-PCR, *AlSRK14* amplification
AlSRK14-R	CAACCCTTCCCACCATCTTGG	RT-PCR, *AlSRK14* amplification
Genomic DNA-F	GGCTGTGACAATGCGAAGCCCC	RT-PCR, genomic DNA contamination
Genomic DNA-R	CCCCATTTTGGTATCTAGGG	RT-PCR, genomic DNA contamination

Supplementary Figure S1

Supplementary Fig. S1. *AlSRK14* and *AlSCR14* transgenic plant selection. *SRK* and *SCR* genes from *A. lyrata* haplotype *S14* were introduced independently into *A. thaliana* Col-0 and C24 accessions, respectively. We determined the Self-Incompatibility (SI) phenotype of transgenic plants by pollination of stage 13-14E stigmas and aniline blue staining. (A) compatibility phenotype: numerous pollen tubes detected in the stigma and style. (B) incompatibility phenotype: less than five pollen tubes detected in the stigma. Bar = 100 μm. (C) SI phenotype of four and two unique insertion lines homozygous for the *AlSRK14* transgene in Col-0 and C24 background, respectively. Pollinations were performed with *A. lyrata S14* pollen. More than 20 pollen tubes were detected in control pollinations (Col-0 x *Al14* or C24 x *Al14*). The exact number of pollen tubes was not determined considering that above 20 pollen tubes the situation is compatible (no error bar). On average, less than five pollen tubes were detected in stigmas of transgenic plants. (D) Quantitative real-time RT-PCR of stage 13-14E stigmas. The level of *AlSRK14* transcripts (with SEM based on three replicates) relative to *ACTIN8 (ACT8)* is compared between two transgenic plants in Col-0 background (#10 and #18) and *A. lyrata S14*. (E) We determined the SI phenotype of five unique insertion lines homozygous for the *AlSCR14* transgene in C24 background. Pollen of these five transgenic plants was deposited on the stigma of the *AlSRK14* line #14 (C24 background). n = number of pollinated stigmas. Pollinations were performed at the same date. Error bars indicate SEM. Dashed line indicates the threshold below which the reaction is considered as incompatible (less than five pollen tubes per stigma).

Supplementary Figure S1

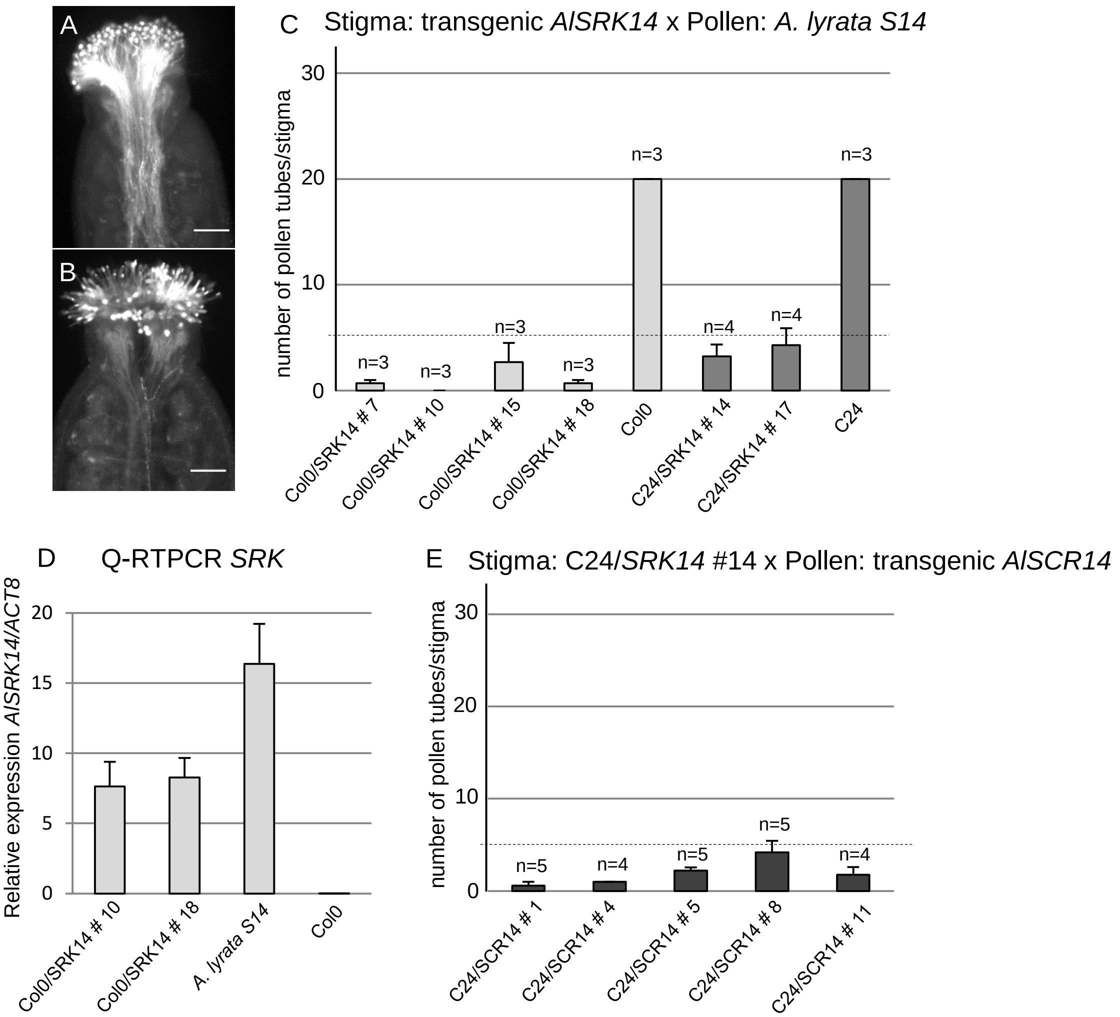

Supplementary table S2

Supplementary table S2. L/W ratio of pollen grains at anthesis

	pollen from anther	
	C24/TURQ	*C24/SCR14+RFP*
mean L/W ratio	2.01	1.92
SEM	0.03	0.03
n	55	49

Supplementary Figure S2

Supplementary Fig. S2. Dynamics of actin focalization following compatible pollination. Examples of the three categories defined in Figure 4B. (A) Actin reorganization in papillae before pollen germination (42 %), (B) during germination (8 %) and (C) after germination (42%). Indicated time corresponds to time after pollen deposition. Images are Z-projections. White Arrow shows the earliest focalization of actin fluorescence. Bar = 10 μm.

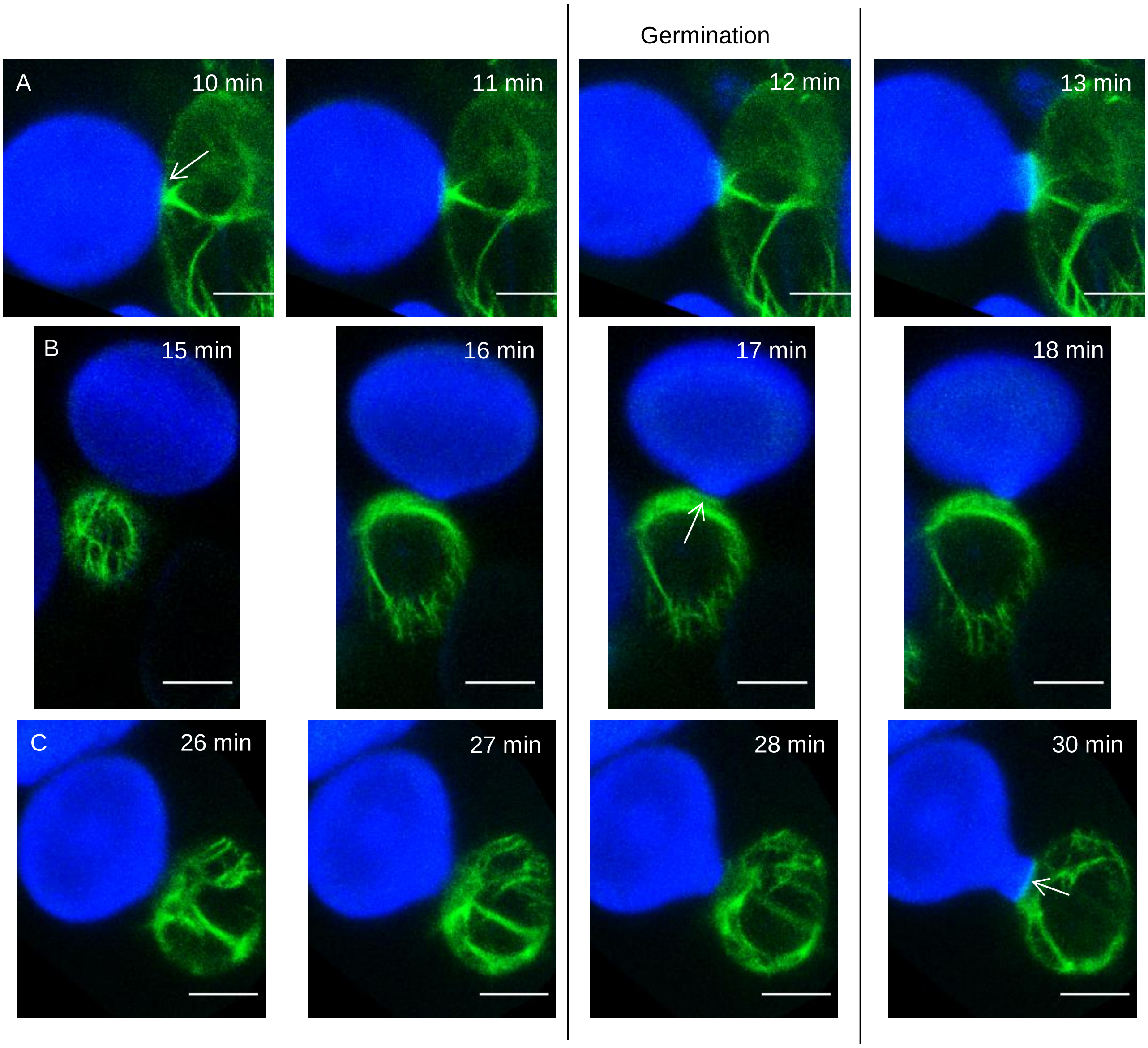

Supplementary Figure S3

Supplementary Fig. S3. Hydration kinetics of compatible and incompatible pollens in the semi *in vivo* system. Images are Z-projections and correspond to enlargement of the pollinated stigma shown in supplementary video S1. Indicated time corresponds to the time after pollen deposition. (A) Length and width (white lines) of one compatible pollen grain. Emergence of a pollen tube (arrow) occurs after 18 minutes. (B) Length and width of one incompatible pollen grain. Bar = 10 μm.

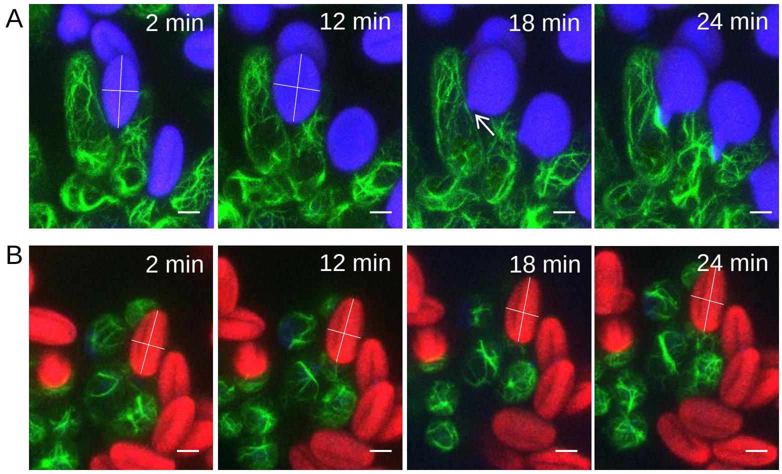

Supplementary table S3

Supplementary table S3. Germination of compatible pollen tracked during experiments described in figure 5. When a pollen grain germinated, it is not anymore included in the L/W ratio calculation

time after pollen deposition	% of grains having germinated	mean L/W ratio
2 min	0%	1.86
4 min	0%	1.76
6 min	0%	1.58
8 min	0%	1.45
10 min	0%	1.39
12 min	5% (3/58)	1.31
14 min	15.5% (9/58)	1.32
16 min	24% (14/58)	1.29
18 min	33% (19/58)	1.28
20 min	43% (25/58)	1.28
22 min	60% (35/58)	1.27
24 min	71% (41/58)	1.25
26 min	84% (49/58)	1.28
28 min	91% (53/58)	1.27
30 min	96.5% (56/58)	1.20
32 min	100% (58/58)	-

Supplementary Figure S4

Supplementary Fig. S4. Standard and high humidity assays. (A) Stigma in contact with the coverslip 30 minutes after pollination in standard conditions. (B) Stigma in contact with the coverslip 30 minutes after pollination in high humidity conditions. Bar = 100 μm

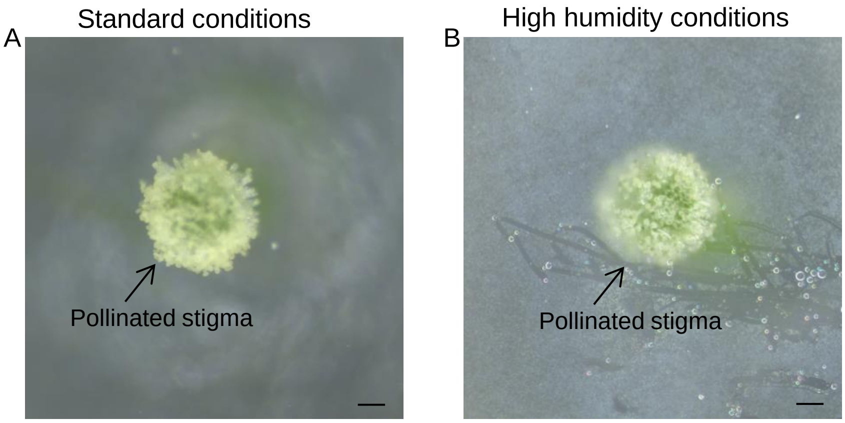

Supplementary Figure S5

Supplementary Fig. S5. Behavior of compatible pollen in high humidity conditions . (A) Features of compatible pollination in high humidity conditions. An Act:Venus marker stigma was pollinated with compatible pollen and incubated in high humidity conditions for 40 minutes. Four independent experiments. (B) Actin (green fluorescence) focalization (white asterisks) in stigmatic cells in contact with blue-fluorescent germinated pollen grains. Image is a single confocal section, 40 minutes after pollen deposition. Bar = 50 μm.

A

% of germination	pollen number
88	586

germination time (min)	SEM	% of papilla exhibiting actin focalization	pollen number
18.55	0.62	75	44

B

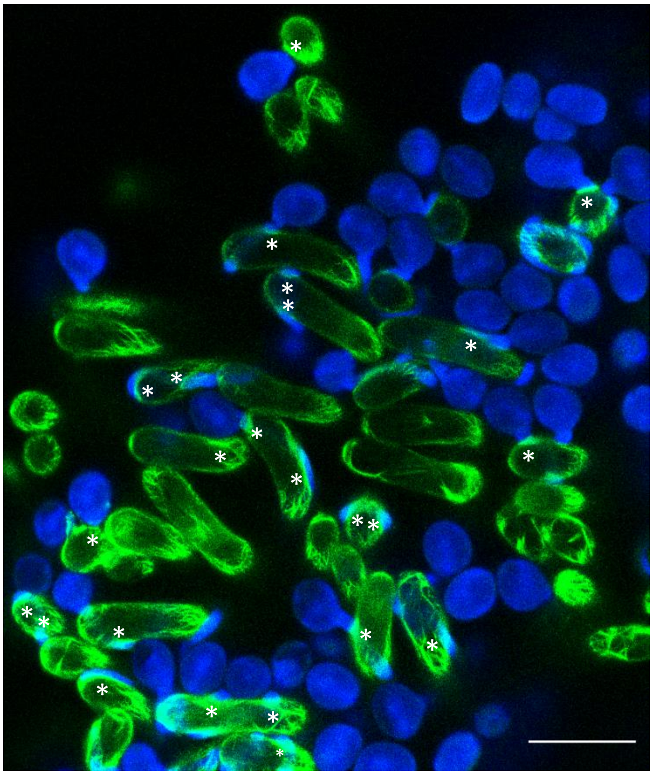

Supplementary Figure S6

Supplementary Fig. S6. Behavior of incompatible pollen in high humidity conditions . (A) Features of incompatible pollination in high humidity conditions. An Act:Venus marker stigma was pollinated with incompatible pollen and incubated in high humidity conditions for 40 minutes. Four independent experiments. (B) Actin (green fluorescence) rarely focalized in stigmatic cells in contact with red-fluorescent germinated pollen grains (only one visible actin focalization: white arrow head). Image is a single confocal section, 40 minutes after pollen deposition. (C) Pollen tubes have an enlarged extremity. Images are Z-projections. Bar = 50 μm

A

% of germination	pollen number
35	314

germination time (min)	SEM	% of papilla exhibiting actin focalization	pollen number
24.87	1.28	13	16

B

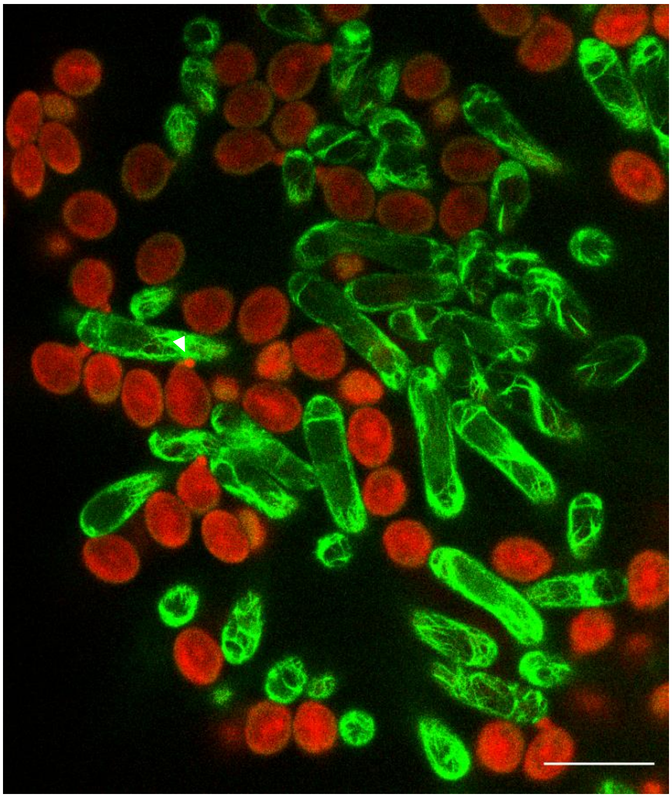

Supplementary table S4

Supplementary table S4. L/W ratio of incompatible pollen in high humidity conditions, 10 minutes after pollen deposition. Highlighted in grey, pollen grains with L/W ratio < 1.4 (21/46 = 46%).

pollen number	L/W ratio at 10 min
1	1.496808511
2	1.571253071
3	1.18293281
4	1.537048014
5	1.706111834
6	1.944564435
7	1.876534789
8	1.918410042
9	1.895803184
10	1.563836682
11	1.266405485
12	1.152023692
13	1.625305623
14	1.500844119
15	1.721281741
16	1.355664488
17	1.092155009
18	1.226078334
19	1.25945658
20	1.305036786
21	1.19333996
22	1.564263323
23	1.338819523
24	1.279553526
25	1.192789969
26	1.338057743
27	1.532069158
28	1.675443968
29	1.376488866
30	1.447643979
31	1.882959369
32	1.323836657
33	1.555009219
34	1.147639956
35	1.26795284
36	1.533791523
37	1.407605785
38	1.527503526
39	1.588938714
40	1.576662144
41	1.302325581
42	1.341981132
43	1.358876117
44	1.405837004
45	1.230807577
46	1.489843297

Supplementary Figure S7

Supplementary Fig. S7. FM4-64 labelling of stigmatic cells. Stigmas were incubated in FM4-64 and imaged under confocal microscopy (red fluorescence). Images are single confocal sections. (A-B) FM4-64 remains at the surface of the stigmatic cells as deduced from its localization above the actin marker Act:Venus (A) and the LTI6b:GFP marker (B) known to label plasma membrane (PM) and endocytic compartments (ec). Bar = 10 μm

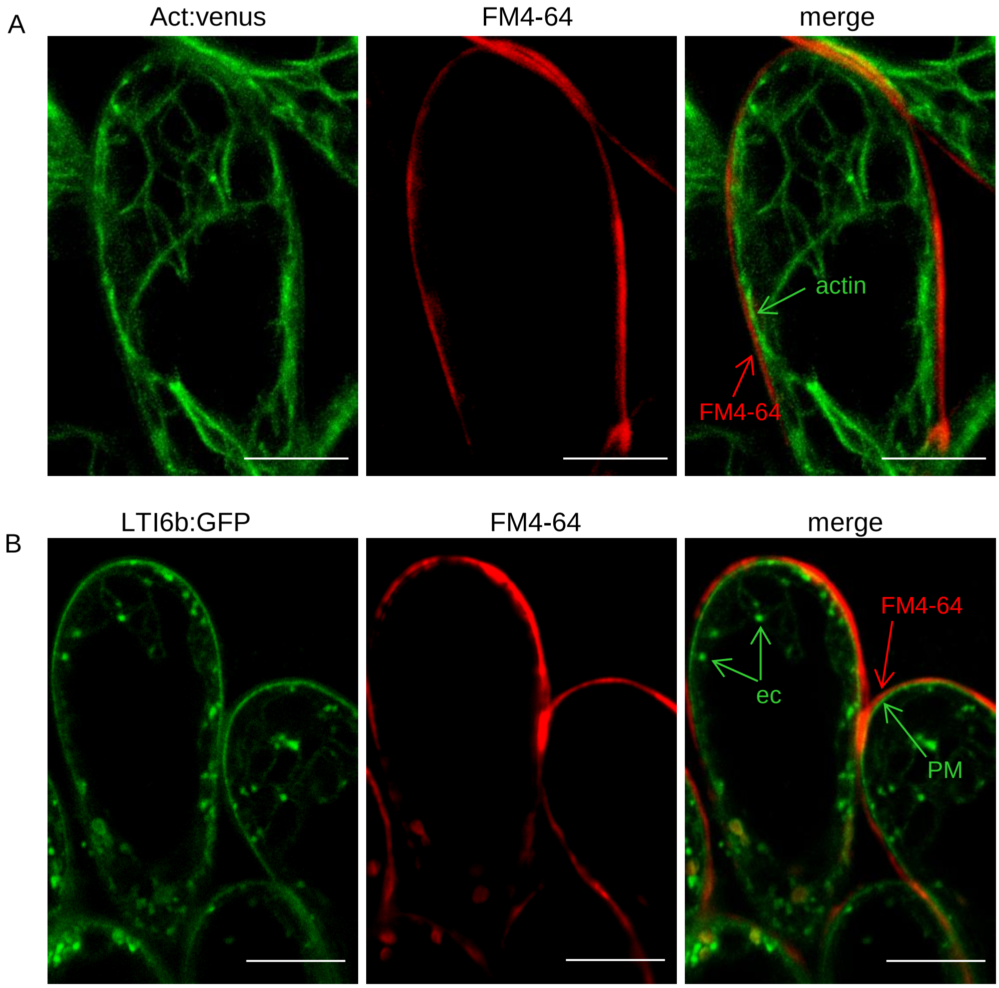

Supplement: eraa008_suppl_supplementary_figures_S1_S7_tables_S1_S4 [file eraa008_suppl_supplementary_figures_s1_s7_tables_s1_s4.pdf]
